# Correspondence

**Published:** 1887-03

**Authors:** G. R. Thomas

**Affiliations:** Pasadina


### CORRESPONDENCE.

PASADINA, January 24, 1887.

DOCTOR TAFT:

Dear Sir: Your letter of the 25th ult. came duly to hand, and gave me much pleasure, I assure you. In my last letter to you I mentioned the fact of having hurriedly examined the implantation case of Dr. Younger's in Dr. Cumming's mouth. Last week I was again in San Francisco, and having more time at my disposal, made it a business to learn all I could of Dr. Younger's operations, and while in the doctor's office I was fortunate in meeting the wife of Dr. H. G. Blankman, one of the pioneer dentists on this coast, and saw the results of operations performed in her mouth last March, and also three days prior to the time of my meeting her. The operation of March last is fully described in a pamphlet of Dr. Younger, which I presume you have seen.

Mrs. B. is a lady well-nigh fifty years old, and the case is that of a left superior bicuspid, and was removed from her own mouth in January, 1885, to relieve neuralgic pain, which was supposed to be caused by the pressure of this tooth. After carrying it in her portemonnaie for a year and two months, she presented herself to Dr. Younger to have it reimplanted, which was done rather reluctantly on the doctor's part, but to-day no person, not even a dentist, would ever suspect that it had ever been removed from its environment.

Three days before I saw her she had the two superior bicuspids on the opposite side reimplanted; one of them had been removed twelve years ago, in an attempt made to remove the other, but was broken off, leaving the root in, which had worked its way to the middle of the space left between the cuspid and first molar, so that it became necessary to remove the root and then drill two new holes on either side of it, making the opening almost one continuous groove.

The teeth were already commencing to get firm, the gum-flaps to contract about the necks, and, withal gave every evidence of a successful termination. A very interesting feature of this case is, that both the replaced teeth have have exostosed roots, which Dr. Younger preferred to use to try to demonstrate the proof of his theory, that the success of the operation depends entirely upon the peridental membrane, a theory which he is very tenacious of, but which is quite the contrary in my own experience of replantation. I was never able to detect any difference in the time or the manner of attachment, whether the peridental membrane had been removed or not, and many times I took a knife and removed every vestige of it, in order to satisfy myself of its value.

Dr. Younger is of the opinion that these implanted teeth in the mouth of Mrs. B. will continue to exostose; will be likely to give future trouble, and probably require removing, which he thinks *will*, or *may*, establish the fact of the necessity of the peridental membrane, which he believes to be the producer of exostosis.

But the question at once arose in my mind as to whether the same constitutional conditions would not become essential in the body of Mrs. B. as did exist in the body of the person in whose mouth those teeth had their exostosed growth. I don't pretend to say that teeth that are devoid of peridental membrane will exostose; but may it not be true, that *other* and unnatural conditions are essential to the production of exostosis? And, again, if the conditions are present in reimplanted teeth sufficient to the production of exostosis (which remains to be proven), are not the conditions such that absorption of the roots, which has troubled us so much in replantation, will be resisted, or, in other words, will not the roots be in condition to resist the absorbing agents, whatever they may be? This question of absorption is one that I have given considerable thought to, and many times when in practice thought I would commence a series of experiments to try and ascertain what the agent was that produces absorption, and then I would be confronted with the fact that I had no time, with the heavy draft made upon me, to brush up my rusty chemical knowledge, what little I ever did have. But now that I have plenty of time, I fully intend, if my health is spared, to enter upon a series of experiments upon the lower animals upon the subject of the replantation of teeth. We have here plenty of rabbits; and Guinea pigs, I suppose, will be accessible. Do you know of any other animals that would be better for such work? And now, my dear doctor, can't or won't you, rather (for I know you can,) give me some points that will enable me to carry on this work systematically, for I do believe that something can be got out of it if persistently followed up.

Yours, most truly and sincerely,

G. R. THOMAS.

